# Morphological and molecular identification of Particolored bat (*Vespertiliomurinus*) in South Korea: A first record

**DOI:** 10.3897/BDJ.12.e135293

**Published:** 2025-01-06

**Authors:** Bong Kyun Kim, Jeong Hun Shim, Sun Sook Kim, Soo Hyung Eo

**Affiliations:** 1 Kongju National University, Yesan, Republic of Korea Kongju National University Yesan Republic of Korea; 2 Chungnam Wild Animal Rescue Center, Yesan, Republic of Korea Chungnam Wild Animal Rescue Center Yesan Republic of Korea; 3 National Institute of Ecology, Seocheon, Republic of Korea National Institute of Ecology Seocheon Republic of Korea

**Keywords:** Chiroptera, mtDNA, morphology, Vespertilionidae, wildlife rescue

## Abstract

Background

*Vespertiliomurinus* belong to the genus *Vespertilio* and are widely distributed in Europe, the Middle East and Northeast Asia, Recently, the presence of the *V.murinus* has been confirmed in Japan, suggesting the possibility of its habitation on the Korean Peninsula. However, ecological information regarding its presence in the Korean Peninsula is extremely limited.

New information

In an urban area of Sejong City, South Korea, a bat within the genus *Vespertilio* was rescued by personnel of the Chungnam Wild Animal Rescue Center. The bat, which was believed to have been hibernating on an exterior wall of a building, was initially identified as *Vespertiliosinensis*. However, the confirmed presence of two pairs of nipples raised the possibility that the bat was a specimen of *V.murinus*. The measurement of the forearm length (FAL) of this bat was 45.67 mm, which is within the 95% confidence interval of the previously reported FALs of *V.murinus*. Additionally, the results of mtDNA sequence analysis indicated that the rescued bat could be differentiated from the closely-related species *V.sinensis* with respect to the sequences of 13PCGs, COI, Cyt*b* and ND1. Finally, phylogenetic analysis revealed that this bat clustered in a clade with previously described *V.murinus*. Collectively, these findings provided convincing evidence to indicate that the rescued individual was a *V.murinus*, marking the first recorded observation of this species in South Korea.

## Introduction

The genus *Vespertilio* (Mammalia, Vespertilionidae) consists of insectivorous bats that primarily inhabit natural structures, such as tree holes, rock crevices and cliffs (Kunz 2013). However, as a consequence of recent habitat loss due to deforestation and other factors, there has been a reported increase in the incidence with which man-made structures are being used as resting sites for these bats (Fukui et al. 2010).

The genus *Vespertilio* comprises two species, viz., the *Vespertiliomurinus* Linnaeus, 1758 and the *Vespertiliosinensis* Peters, 1880, both of which have been observed or inhabit the Korean Peninsula (Simmons and Cirranello 2024). *V.murinus* is widely distributed in Europe, the Middle East, Northeast Asia and the high-latitude regions of the Korean Peninsula (Brabant et al. 2016, Jo et al. 2018). The species was first discovered in Japan in 2002 (Satô and Maeda 2003), and since then, a further three individuals were observed in 2005 (Kawai et al. 2010). In 2011, a group including offspring was observed, thereby confirming that these bats inhabit Japan year-round (Kondo et al. 2012, Kawai et al. 2015). There have been very few observations of this species in North Korea and, to the best of our knowledge, no reports from South Korea (Jo et al. 2018). Comparatively, *V.sinensis* has a relatively narrow distribution range concentrated within the Korean Peninsula, Mongolia, Japan, China and Russia (Fukui and Agetsuma 2010).

The two *Vespertilio* species are characterised by a similar external appearances, thereby making it particularly difficult to distinguish one from the other based on a visual inspection alone. However, compared with *V.sinensis*, *V.murinus* has been established to have a slightly smaller body size (Jo et al. 2018). Furthermore, these species differ in terms of the number of nipples present, making this a useful distinguishing feature (Kondo et al. 2012, Safi 2023). Whereas *V.murinus* has two pairs of nipples (four in total), *V.sinensis* has a single pair (two in total). However, these nipples are extremely small in size and generally well concealed (Haarsma 2008) and their size can differ depending on factors such as stage of growth, sex or pregnancy (Simmons 1993). Consequently, without a close examination of specimens, the number of nipples is often difficult to determine and, thus, the use of alternative indicators that show species specificity, such as the length of each part of the skull and forearm, is considered necessary for facilitating accurate identification (Kawai et al. 2010).

Between 2010 and April 2023, a total of 61 bats in the genus *Vespertilio* were rescued by the Chungnam Wild Animal Rescue Center (CNWARC), located in the central-western region of South Korea. Amongst these, an individual rescued in Sejong City in December 2022 was assumed to be a specimen of *V.murinus*, based on confirmation of the presence of two pairs of nipples. This finding accordingly provided evidence to indicate that the migration route or habitat distribution of *V.murinus* could include part of the Korean Peninsula (Satô and Maeda 2003). In this study, we thus sought to confirm the identity of this bat using morphological and molecular tools. To the best of our knowledge, this study is the first to record *V.murinus* in South Korea and our findings will provide genetic information on species of the genus *Vespertilio* that inhabit South Korea.

## Materials and methods

**Morphology**

**Figure 1.**
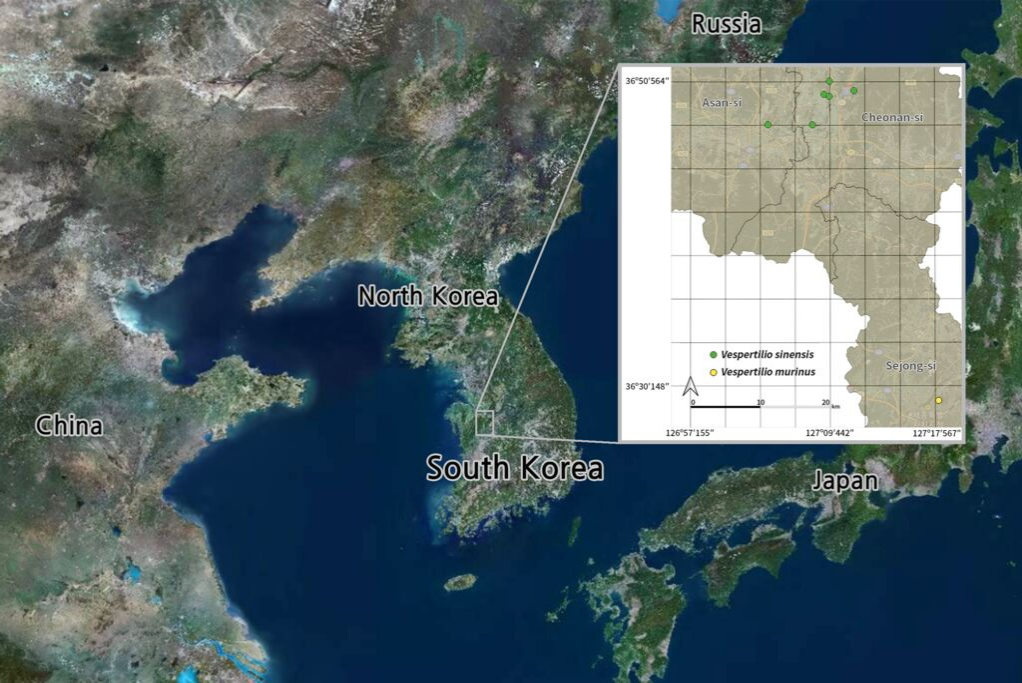
Collection localities for genus *Vespertilio* bats in South Korea.

We collected bats of the genus *Vespertilio* in Chungcheongnam-do, South Korea (Fig. 1). We then conducted external morphological measurements to identify their species, determine inter-species differences and obtain basic information related to bats of the genus *Vespertilio*. The bats examined included live individuals and, accordingly, we adopted previous procedures that can be used on live bats (Kawai et al. 2010, Chung et al. 2015). Thus, we measured the forearm length (FAL) and body weight (BW) of the bats (Table 1). FAL was measured using Vernier calipers (unit 0.01 mm; Mitutoyo, Kanagawa, Japan) and body weight was measured using an electronic scale (unit 0.01 g; ACOM Co., Ltd, Tokyo, Japan). However, given the considerable variability in body weight, even in the same individual depending on the time of measurement, such as during the active period or the beginning and end of the hibernation period, this can lead to a lack of uniformity in the collected weight-related data (Ryu et al. 2024). Thus, we excluded weight measurements from the final analysis. Indeed, the individuals analysed in this study were rescued in a weakened state due to accidents and it was accordingly assumed that the measured weights could be significantly lower than those of healthy individuals.

**DNA sequencing and molecular analysis**

For the purposes of molecular analyses, we extracted DNA from tissues samples obtained from six bats. A bat presumed to be a *V.murinus* was kept alive to minimise physical damage and side effects that could occur during the sample collection process. A tissue biopsy was performed from the wing membrane using a 4 mm biopsy punch (KAI MEDICAL, Saeki, Japan). The remaining five bats were dead and, consequently, samples were collected from the pectoral muscles. Extraction of genomic DNA was performed using a DNeasy Blood & Tissue Kit (QIAGEN, Hilden, Germany). The samples collected from the bats and the extracted DNA were stored at Kongju National University.

Sequences of 13PCGs, COI, Cyt*b* and ND1 regions were analysed to identify the suspected *V.murinus* at the species level. For amplification of 13PCGs, next-generation sequencing (NGS) was performed using a Novaseq6000 system (Macrogen Inc., Seoul, Korea). The COI, Cyt*b* and ND1 regions were amplified using the jgl.CO1490 and jgHCO2198 (Jargalsaikhan et al. 2022), 13ed2 and BAT15Red2 (Kawai et al. 2003) and ND1F2ed and ND1R2ed (Kawai et al. 2002) primer pairs, respectively, with slight modifications made as needed. PCR was performed to amplify each genetic region according to the protocol recommended for A-star Taq DNA polymerase (BIOFACT, Daejeon, Korea) and sequence editing was performed using GENEIOUS v.10.2.5 software (Kearse et al. 2012).

The selection of bat species used to determine phylogenetic relationships was based on a previous study (Agnarsson et al. 2011) and the classification system followed that of Bat Species of the World (Simmons and Cirranello 2024). The phylogenetic trees constructed for the respective gene sequences included the base sequences from species registered in the GenBank database (National Center for Biotechnology Information), as well as those of the six *Vespertilio* bats analysed in this study (Suppl. material 1). Trees were constructed using the Maximum-Likelihood (ML) algorithm in PhyML v.3.0 (Guindon et al. 2010). The best models for calculating nucleotide substitutions were determined using Smart Model Selection (Lefort et al. 2017) and the reliability of each phylogenetic tree was tested using 1,000 bootstrap replicates (Felsenstein 1985).

Inter- and intra-species differences amongst haplotypes were verified using the COI base sequence information obtained for the six *Vespertilio* bats and base sequence information for *V.murinus* and *V.sinensis* was obtained from GenBank. A TCS network analysis using Popart 1.7 software (http://popart.otago.ac.nz) was run to determine the genes at a population level. Additionally, to confirm the relative genetic distance between the *Vespertilio* species, we compared the sample size (N), number of haplotypes and haplotype diversity.

## Results

**Morphological analysis**

**Figure 2.**
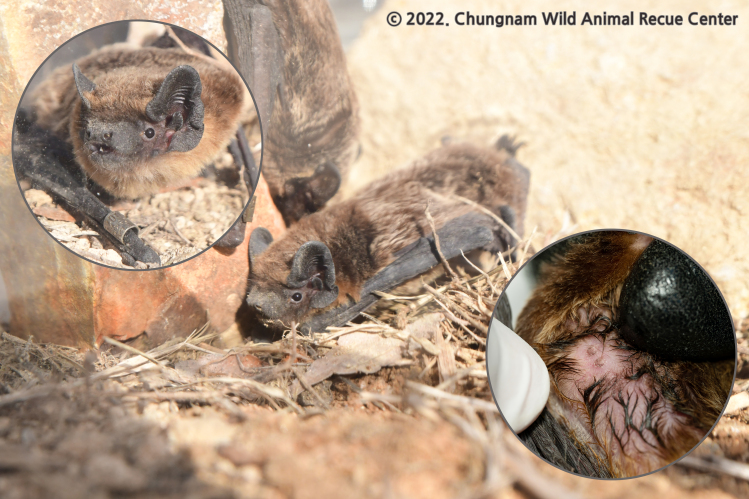
Morphological characteristics of the *Vespertiliomurinus* collected by the CNWARC in 2022. It has a pair of nipples (four in total).

**Figure 3.**
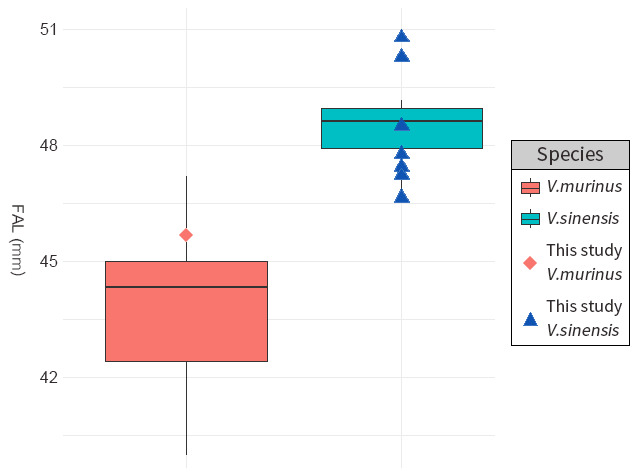
Scatter plot and box plot of the forearm length of genus *Vespertilio* collected in this study and previous studies.

We measured the FAL of a total of nine bats in the genus *Vespertilio*, one of which was presumed to be a specimen of *V.murinus* (Fig. 2). The FAL of this individual was 45.67 mm, which compares with the average and standard deviation values of 44.07 mm (± 1.91 mm) obtained for the FAL of *V.murinus* in previous studies (Table 2). Comparatively, the average FAL of the eight *V.sinensis* individuals was 48.28 mm (± 1.50 mm), which is almost identical to the previously reported value of 48.29 mm (± 1.11 mm) obtained for this species (Table 2). Thus, the FAL of the *V.murinus* individual examined was somewhat shorter than the measured values obtained for *V.sinensis* in the present and previous studies (Fig. 3).

**Phylogenetic tree analysis**

**Figure 4.**
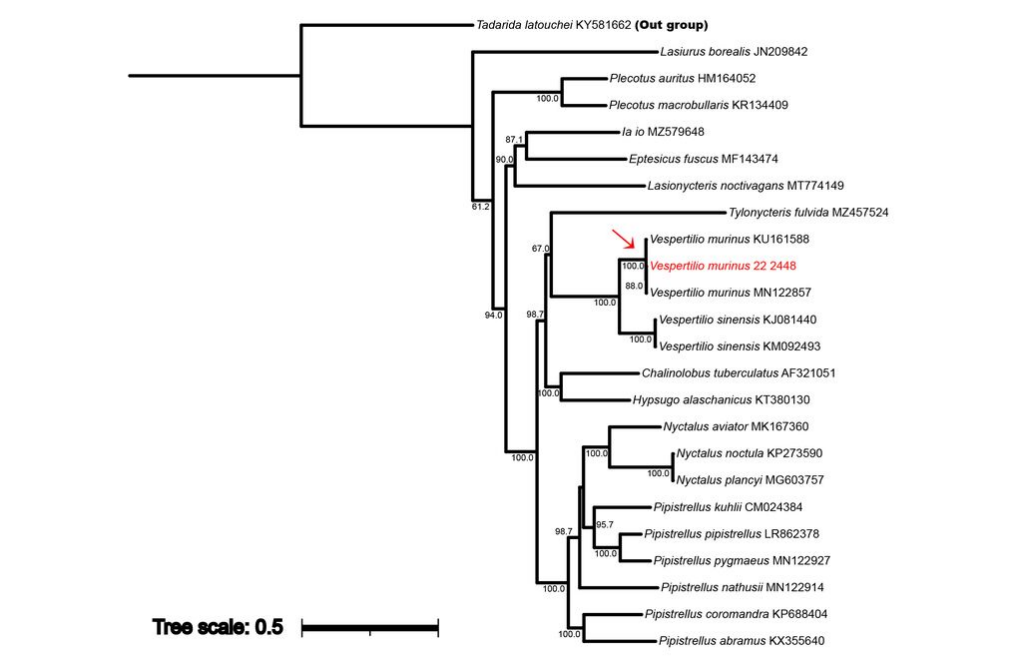
Maximum Likelihood tree based on 13 mitogenomic protein-coding genes of the *Vespertiliomurinus* using TN92+R. Bootstrap percentages of more than 50% are shown. *Tadaridalatouchei* was designated as the outgroup.

**Figure 5.**
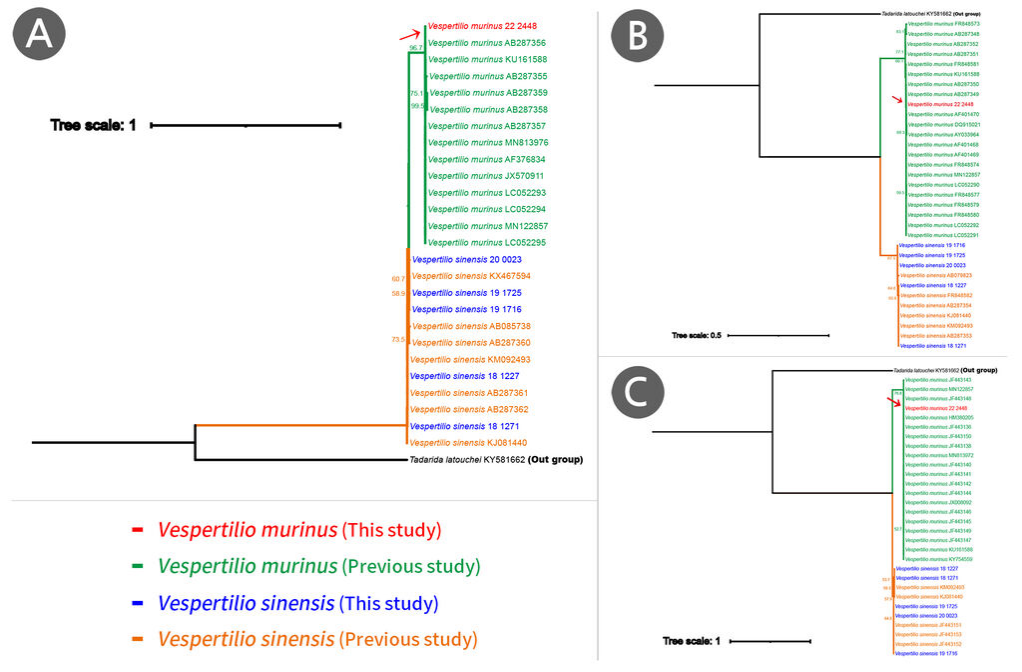
Phylogenetic tree for genus *Vespertilio*, constructed using the Maximum Likelihood method. *Tadaridalatouchei* was designated as the outgroup. Analysis based on A: Cyt*b* gene; B: ND1 gene; C: COI gene.

Amongst the six *Vespertilio* bats subjected to molecular analysis, the specimen presumed to be *V.murinus* was observed to cluster in same clade as other *V.murinus* registered in the NCBI database with 100% bootstrap support in the ML phylogenetic tree constructed, based on 13PCGs (Fig. 4). Consistently, it also grouped in the same clade as other *V.murinus* in trees, based on the sequences of COI, Cyt*b* and ND1 regions (Fig. 5). Moreover, based on the 13PGCs, we obtained genetic distances of between 0.08 and 0.29 (%) between the *V.murinus* examined in this study and the GenBank *V.murinus* accessions, whereas genetic distances of 11.31 to 11.32 (%) were obtained between this individual and the *V.sinensis* (Table 3). Similarly, for all analysed genetic regions, the five individuals of *V.sinensis* examined in this study formed a single clade with other *V.sinensis* listed in the NCBI database (Fig. 5).

**Haplotype network analysis**

**Figure 6.**
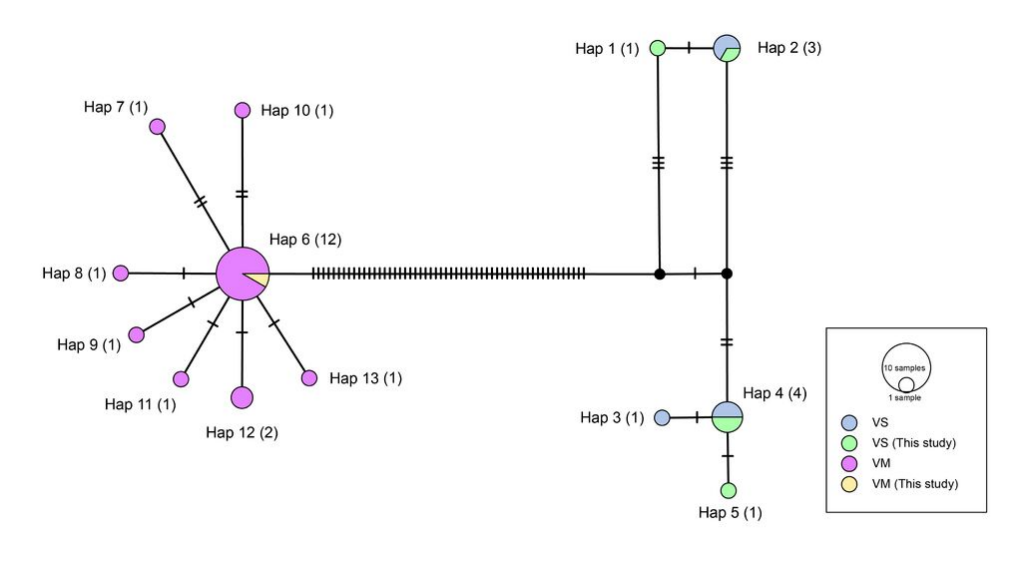
Haplotype network of genus *Vespertilio* based on COI. The size of circle represents number of haplotypes (VS: *Vespertiliosinensis*, VM: *Vespertiliomurinus*).

Analysis of the COI base sequences of the two species revealed that the haplotypes of *V.murinus* and *V.sinensis* were clearly distinguished at the species level, although were not as clearly distinguished at the intra-species and habitat levels. *V.murinus* formed a radial pattern centred on Hap 6 (12 individuals) (Fig. 6) and we confirmed that the individual analysed in this study also had a Hap 6. The *V.sinensis* had the most individuals with Hap 4 (four individuals) and we confirmed that the five individuals analysed in this study had different haplotypes, apart from for two individuals that both had the Hap 4 (Fig. 6).

## Discussion

According to morphological and genetic data, an individual of the genus *Vespertilio* rescued in South Korea was identified as *V.murinus*. The measured FAL was compared with those recorded in Europe (Baagøe 2001, Baranauskas et al. 2006, Murariu 2007, Alberdi et al. 2012), Japan (Satô and Maeda 2003, Kawai et al. 2010, Kawai et al. 2015), Iran, the United Arab Emirates (Benda et al. 2012) and South Korea (Chung et al. 2015). The FAL of the *V.murinus* we collected fell within the confidence intervals of FAL lengths reported in multiple previous studies, although it was higher than the average length. We believe this result is due to the fact that multiple individuals measured the FAL, variables such as sex and growth stage were not considered and the collected information was somewhat limited. Nevertheless, since the FAL length of *V.murinus* falls outside the confidence interval of measurements for *V.sinensis*, we concluded that the individual we collected corresponds to *V.murinus*.

Genetic analyses are effective tools for identification of species (Herbert et al. 2003) and, on the basis of the analysis of multiple genetic regions, we obtained convincing evidence to indicate that the suspected *V.murinus* did indeed differ from *V.sinensis* at the species level. This accordingly substantiates the superiority of mtDNA barcoding methods over a dependence on external morphology with respect to species identification and, thereby, highlights its utility for establishing management and conservation measures for different taxa. On the basis of an analysis of the 13PCG region, we obtained genetic distances of 0.08 and 0.29 (%) between the *V.murinus* examined in this study and those collected from Denmark and Iran, respectively. Given that Iran is geographically closer to South Korea than Denmark, we anticipated that the examined *V.murinus* individual would show a closer genetic relationship to conspecifics from Iran, rather than to those from the more distant Denmark.

This study offers three possible explanations for the appearance of the *V.murinus* in South Korea. The first possibility is that the migratory *V.murinus* could arrive or pass through South Korea at a specific time. The *V.murinus*, a relatively small part of the population from the north-western part of the European summer distribution area, is known to migrate south and south-west in the autumn, including along the North Sea coast, sometimes over long distances, up to more than 1,500 kilometres (Pauza and Pauziene 1998, Ahlén et al. 2009, Brabant et al. 2016). The distinction between dispersion and migration is not always clear, but the spikes in the number of observations in Belgium in the spring (April/May) and especially in the autumn (mid-August to mid-October) and a very calm period from late May to mid-August, seem to be a strong indication for a migration pattern (Brabant et al. 2016). *V.murinus* may also migrate along the coast or over the sea and, accordingly, it is possible that stray bats would appear occasionally on the island and other localities nearby (Kawai et al. 2015). Additionally, it could be a natural event that occurred during the long-distance movement or migration. Several bat species, including the *V.murinus*, have often been observed unexpectedly in areas due to natural events, such as typhoons or during their movement or migration process (Pauza and Pauziene 1998, Ahlén et al. 2007). In a previous study on *V.murinus* observed in Japan, it was suggested that individuals living on the Asian continent may have been accidentally introduced to Japan by the strong winds encountered during the migration process (Kawai et al. 2010). The second possibility is that the *V.murinus*, like the *V.sinensis*, may be gradually moving or spreading from high-to low-latitude areas. Changes in the environment can act as a pressure to promote the movement of animals, depending on specific species, taxonomic groups and geographical characteristics (Shaw 2016). Climate change, including global warming, is known to have direct and indirect effects on the decrease in the number of individuals of certain animal species and changes in habitat distribution and migration (Howard et al. 2024). Therefore, the observation of a species that was relatively common in high-latitude areas in low-latitude areas suggests the possibility that these species may gradually move or spread due to changes in climate and other environments. If records accumulate over time, the *V.murinus* will be observed more frequently and widely in South Korea and it is expected that weight will be placed on this possibility. The third possibility is that bats may have been inadvertently included in air travel, ports and cargo and, thus, artificially moved as previous studies have confirmed this (Constantine 2003, Ruffell et al. 2009). Cheongju International Airport is approximately 30 km from the location where the *V.murinus* was observed. Cheongju International Airport operates direct flights to China (Yanji) and Japan (Tokyo, Osaka, Fukuoka), where the *V.murinus* is known to inhabit; therefore, there is a possibility that the individual investigated in this study was introduced via an aeroplane or air cargo. The additional possibility is that *V.murinus* has already been residing in South Korea at some point in the past. *V.murinus* is known to inhabit a wide range of the Eurasian continent, excluding the Polar regions, but has rarely been reported in the Far East (Jo et al. 2018). However, the possibility that this species is established in the Far East was suggested when it was confirmed to reside year-round in Japan (Kondo et al. 2012, Kawai et al. 2015). Given the lack of relevant research and opportunities to confirm its presence, it is conceivable that the presence of *V.murinus* in South Korea has hitherto been overlooked. Indeed, the two species of *Vespertilio* tend to utilise similar habitats and inhabit similar environments, including villages, montane forests and riparian areas (Jo et al. 2018). Accordingly, it is plausible that *V.murinus* already has a foothold in South Korea, although this has not been recognised due the close anatomical, behavioural and ecological characteristics of *V.murinus* and *V.sinensis*.

Given that bats are the only mammals with the natural ability to fly, they are not limited to such a great extent as are terrestrial mammals by geographical barriers and can potentially traverse long distances (O’Shea et al. 2014). Although bats have been identified as vector species that can transmit a number high-risk infectious diseases, including rabies and the Covid virus (Ma et al. 2020), they are also considered to play a number of beneficial roles, in that they perform ecological functions essential to humans, such as pest control, moisture mediation and seed dispersal (Scott et al. 2011). Consequently, the findings of this study highlight the importance of the appropriate management of bats, which is necessary to prevent the spread of infectious diseases, maintain public health safety, secure food resources and promote agriculture. Moreover, these findings will also contribute to the maintenance of ecosystem health and diversity. Accordingly, more effort is required to study and evaluate the ecological characteristics and roles of bats, as well as developing sustainable management measures.

## Supplementary Material

E1193EBB-C490-5801-A2BA-2E5835AD5996 10.3897/BDJ.12.e135293.suppl1 Supplementary material 1 Collection Datasheet Data type Collection and sampling details, genetic data. File: oo_1202108.xlsx https://binary.pensoft.net/file/1202108 Kim, B. K., Shim, J. H., Kim, S. S., and Eo, S. H.

## Figures and Tables

**Table 1.** Location and measurements data of the genus *Vespertilio* from South Korea. (CNWARC: Chungnam Wild Animal Rescue Center, NIE: National Institute of Ecology). *BW was measured at the time of receipt of each object and may have been indicated depending on the measurer. **FAL was measured by the same person to reduce errors.

**Number**	**Sepecies**	**Date**	**Latitude, Longitude**	**Sex**	BW* (g)	FAL** (mm)	**Collector**
14-0707	*V.sinensis*	14. 12. 08.	36°50'03.8", 127°08'40.2"	F	14	46.67	CNWARC
18-1227	*V.sinensis*	18. 12. 03.	36°50'24.7", 127°11'06.0"	M	16.14	47.78	CNWARC
18-1271	*V.sinensis*	18. 12. 21.	36°50'03.8", 127°08'40.2"	M	16	47.49	CNWARC
19-1716	*V.sinensis*	19. 12. 12.	36°48'03.8", 127°03'37.5"	F	16.4	47.43	CNWARC
19-1725	*V.sinensis*	19. 12. 16.	36°48'04.4", 127°07'30.1"	M	17.49	48.51	CNWARC
20-0023	*V.sinensis*	20. 01. 08.	36°51'03.7", 127°08'56.3"	F	14	47.25	CNWARC
23-0051	*V.sinensis*	23. 01. 14.	36°46'24.7", 127°08'00.1"	F	27.65	50.30	CNWARC, NIE
23-0062	*V.sinensis*	23. 01. 21.	36°55'28.1", 127°03'19.5"	F	22.50	50.79	CNWARC, NIE
22-2448	*V.murinus*	22. 12. 15.	35°28'58.4", 127°18'16.2"	F	17.04	45.67	CNWARC, NIE

**Table 2.** Location and measurements data of the genus *Vespertilio* collected from previous studies and this study.

Number	Species	Sex	BW (g)	FAL (mm)	Locality	Reference
-	*V.murinus*	-	-	44.20	-	Baagøe (2001)
-	*V.murinus*	M	-	45.00	Vilnius, Lithuania	Baranauskas et al. (2006)
MAM9724	*V.murinus*	F	12	42.00	Bucharest, Romania	Murariu (2007)
MAM6200	*V.murinus*	M	-	40.00	Baia, Romania	Murariu (2007)
MAM5884	*V.murinus*	M	-	42.00	Zãrneºti, Romania	Murariu (2007)
KK162	*V.murinus*	M	10	44.80	Chitose, Hokkaido, Japan	Kawai et al. (2010)
KK163	*V.murinus*	M	11.6	44.20	Haboro, Hokkaido, Japan	Kawai et al. (2010)
KK164	*V.murinus*	M	8.5	46.00	Minmaya, Aomori, Japan	Kawai et al. (2010)
RTMM188	*V.murinus*	F	12.1	45.00	Kahuka, Hokkaido, Japan	Kawai et al. (2010)
-	*V.murinus*	-	-	44.20	Bielsa, Spain	Alberdi et al. (2012)
-	*V.murinus*	-	-	46.80	Bielsa, Spain	Alberdi et al. (2012)
-	*V.murinus*	-	-	47.20	Bielsa, Spain	Alberdi et al. (2012)
-	*V.murinus*	-	-	42.50	UAE	Benda et al. (2012)
-	*V.murinus*	-	-	42.20	Iran	Benda et al. (2012)
-	*V.murinus*	-	-	44.50	Iran	Benda et al. (2012)
YT080923	*V.murinus*	M	-	44.50	Hegura-jima, Hokkaido, Japan	Kawai et al. (2015)
22-2448	*V.murinus*	F	17.04	45.67	Sejong, Korea	This study
-	*V.sinensis*	-	-	48.90	-	Baagøe (2001), Satô and Maeda (2003)
-	*V.sinensis*	-	20	48.36	Jeongeup, Jeonbuk-do, Korea	Chung et al. (2015)
-	*V.sinensis*	-	22.4	49.18	Jeongeup, Jeonbuk-do, Korea	Chung et al. (2015)
-	*V.sinensis*	-	21.1	46.70	Jeongeup, Jeonbuk-do, Korea	Chung et al. (2015)
14-0707	*V.sinensis*	F	14	46.67	Cheonan, Chungnam-do, Korea	This study
18-1227	*V.sinensis*	M	16.14	47.78	Cheonan, Chungnam-do, Korea	This study
18-1271	*V.sinensis*	M	16	47.49	Cheonan, Chungnam-do, Korea	This study
19-1716	*V.sinensis*	F	16.4	47.43	Asan, Chungnam-do, Korea	This study
19-1725	*V.sinensis*	M	17.49	48.51	Cheonan, Chungnam-do, Korea	This study
20-0023	*V.sinensis*	F	14	47.25	Cheonan, Chungnam-do, Korea	This study
23-0051	*V.sinensis*	F	27.65	50.30	Cheonan, Chungnam-do, Korea	This study
23-0062	*V.sinensis*	F	22.5	50.79	Asan, Chungnam-do, Korea	This study

**Table 3.** Genetic distances between individual bats of the genus *Vespertilio* analysed through 13PCGs (VM: *Vespertiliomurinus*, VS: *Vespertiliosinensis*). Below diagonal: % of genetic similarity. Above diagonal: % of genetic difference.

	VM 22-2448 (This study)	VM KU161588	VM MN122857	VS KJ081440	VS KM092493
VM 22-2448 (This study)	-	0.29	0.08	11.32	11.31
VM KU161588	99.71	-	0.29	11.36	11.31
VM MN122857	99.92	99.71	-	11.32	11.31
VS KJ081440	88.68	88.64	88.68	-	0.2
VS KM092493	88.69	88.69	88.69	99.80	-
